# The Effectiveness of Levamisole and Broccoli in Lead Poisoning: Hematobiochemical Changes and Tissue Damage in the Liver, Kidney, and Spleen of Wistar Rats

**DOI:** 10.1155/jt/8283897

**Published:** 2024-11-29

**Authors:** Kimia Shahbazi, Mahdieh Raeeszadeh, Loghman Akradi

**Affiliations:** ^1^Faculty of Veterinary Sciences, Sanandaj Branch, Islamic Azad University, Sanandaj, Iran; ^2^Department of Basic Sciences, Sanandaj Branch, Islamic Azad University, Sanandaj, Iran; ^3^Department of Pathobiology, Sanandaj Branch, Islamic Azad University, Sanandaj, Iran

**Keywords:** broccolini, lead, levamisole, liver, rat, spleen

## Abstract

Lead, a heavy metal, has emerged as one of the most significant pollutants, bearing irreversible consequences on human and animal health in conjunction with industrial development. Presently, the use of medicinal plants to alleviate the adverse effects of heavy metal toxicity has captured the attention of researchers. Hence, the objective of this study was to assess the impact of levamisole and broccoli extract on the electrophoretic pattern of serum proteins, hematological parameters, and histopathological alterations in the liver, kidney, and spleen tissues within a lead poisoning model of rats. This experimental investigation spanned 28 days, involving 42 male Wistar rats categorized into seven groups: a control group, a lead acetate (AL) group administered at 1000 ppm in drinking water, a broccoli (B) group at 300 mg/kg/day, a levamisole (LE) group at 2.5 mg/kg/day, and combination groups of lead and broccoli (AL + B), lead and levamisole (AL + LE), and lead, broccoli, and levamisole (AL + LE + B). Upon completion of the study, hematological and biochemical parameters were assessed, and serum protein concentrations were analyzed using electrophoresis. Liver, kidney, and spleen tissues were fixed and subjected to histopathological examination with H&E staining. The findings indicated a significant decrease in white blood cells (WBC), red blood cells (RBC), and hemoglobin (Hb) levels in the AL group compared to other groups (*p* < 0.01). Conversely, the B group exhibited a notable increase in RBC and WBC compared to the AL group (*p* < 0.05). The most pronounced lead-induced damage was observed in the liver, resulting in elevated levels of specific enzymes such as AST and ALT in the AL group, accompanied by a decline in albumin and total protein (*p* < 0.001). A reduction in globulin levels, including Beta-2 globulin, was noted in the AL + B and AL + LE groups compared to the AL group (*p* < 0.001,  *p* < 0.05). Histopathological findings also unveiled increased infiltration of inflammatory cells and hemorrhage in the liver tissue, followed by the spleen, significantly higher in the AL group compared to other experimental groups (*p* < 0.05). Additionally, congestion and inflammation were evident in the spleen tissue compared to other groups. These tissue damages were mitigated in other combination treatment groups. Based on the aforementioned results, the combination of broccoli and levamisole is deemed effective in ameliorating liver and spleen injuries caused by lead and enhancing biochemical parameters and serum proteins.

## 1. Introduction

Lead, considered one of the most toxic heavy metals for human health, inflicts damage on neural connections and leads to blood and brain disorders [1]. Consequently, researchers have consistently endeavored to eliminate lead from the environment. The primary mechanism of heavy metal toxicity in the body is oxidative stress induction. Lead induces inflammation, stimulating the immune system and consequently increasing the number of white blood cells (WBC), especially lymphocytes, in the blood [2]. Past studies have reported a significant decrease in platelet count in lead-exposed groups compared to control groups. In contrast, Okon et al. observed an increase in platelets after administering lead acetate to adult male rats [3]. The exact mechanism of lead action on platelet count has not yet been identified. Some believe that lead causes lipid membrane peroxidation and increases reactive oxygen species (ROS) in platelets, leading to their destruction [4].

Furthermore, lead, by damaging red blood cells (RBC) and disrupting liver and kidney function, inhibits erythropoietin secretion from these organs' specialized cells, thereby reducing their numbers in the blood [5]. Additionally, a decrease in hematocrit and hemoglobin (Hb) percentage can be correlated with a decrease in RBC count [6].

Oxidative stress results in the accumulation of significant amounts of lead in the liver, leading to oxidative stress induction [7]. This results in the simultaneous inhibition of several antioxidant enzymes such as superoxide dismutase (SOD), catalase, glutathione (GSH) peroxidase, GSH reductase, and the concomitant increase in GSH disulfide and reduction in the GSH/GSSG ratio [8].

The concept that bioactive components in functional foods contribute to health improvement has recently gained importance [9].

Cruciferous vegetables such as broccoli, cabbage, and cauliflower are excellent sources of phytochemicals such as glucosinolates and their by-products, phenolics, and antioxidant vitamins, as well as minerals in the diet [10]. Consumption of broccoli mediates various functions, including providing antioxidants, regulating enzymes, and controlling apoptosis and the cell cycle. Organosulfur compounds, namely glucosinolates and S-methyl cysteine sulfoxide present in broccoli, along with other compounds such as vitamins K, C, E, and minerals like iron, zinc, selenium, and polyphenols such as kaempferol, quercetin glucosides, and isorhamnetin, may be responsible for the various health benefits of broccoli [11].

A study published in 2014 demonstrated that broccoli has strong antioxidant and anti-inflammatory effects [12]. Broccoli's vitamin K1 content includes significant amounts of K1, which is important for blood clotting [13]. Sulforaphane is an antioxidant with many health benefits. It can protect against heart disease, cancer, inflammation, and allergies [14]. Broccoli, rich in iron and protein, therefore provides an excellent remedy for anemia. Broccoli's vitamin C is an important antioxidant for immune function and skin health. Additionally, broccoli is abundant in flavonoids that have anti-inflammatory and antioxidant effects [15].

Levamisole, a low-molecular-weight synthetic compound, is the first member of a new class of drugs that can enhance cellular immunity in healthy laboratory animals [16]. The properties of levamisole help in better understanding molecular events that trigger immune responses. Levamisole can act as an immune system stimulant with an immune system suppressant and growth promotion [17, 18]. These effects depend on the dose administered, the time of administration, the experimental method used to measure the impact, and the host's genetic background [19].

Given the reports from researchers indicating that lead poisoning weakens the immune system and induces oxidative stress, and considering the immunomodulatory and antioxidant potentials of levamisole and broccoli, the aim of this study was to evaluate the effects of levamisole and broccoli on lead poisoning in rats. This investigation focused on assessing hematological indices, liver and kidney biochemical parameters, and changes in serum protein patterns.

## 2. Materials and Methods

### 2.1. Study Animals

An experimental intervention study was conducted on 42 (200–250 g, 6–8 weeks old) male Wistar rats. The animals were kept in the Animal House at the Islamic Azad University, Sanandaj Branch, under controlled environmental conditions, including a temperature of 20 ± 2°C, humidity of 50%–55%, and a 12-h light/dark cycle. They had ad libitum access to clean water and rodent feed. The study commenced after a 7-day acclimatization period to these conditions. Principles of laboratory animal care were followed based on international standard guidelines, and approval was obtained from the Ethics Committee in Medical Sciences of the Islamic Azad University, Sanandaj Branch, with code IR.IAU.SDJ.REC.1401.029.

### 2.2. Preparation of Broccoli Methanol Extract

After obtaining the broccoli plant and its verification by the Medicinal Plants Research Center of Kurdistan University, it was dried and milled. Fifty grams of broccoli powder was soaked in 250 mL of 80% methanol alcohol by the maceration method for 48 h. During this period, a shaker was used to ensure the complete dissolution of extract compounds in alcohol. After filtering the extract, it was placed in a rotary evaporator under vacuum to evaporate the alcohol. Finally, a dosage of 300 mg/kg was considered for administration.

### 2.3. Animal Grouping

The animals were randomly divided into 6 groups of 7 each and subjected to treatment:

  Control group: received standard water and diet without treatment.
  AL group: received lead acetate at a dose of 1000 ppm in water [20].
  B Group: received oral broccolini methanol extract at a dose of 300 mg/kg of body weight once a day [21].
  LE group: received oral levamisole at a dose of 2.5 mg/kg of body weight once a day [22].
  AL + B group: received lead acetate in water and broccoli extract at the previous doses.
  AL + LE group: received lead acetate in water and levamisole at the previous doses.
  Combined group (AL + LE + B): received lead acetate in water, broccoli extract, and levamisole at the same doses as the previous groups.

The animals were treated for 28 days according to their assigned groups. At the end of the treatment period, blood samples were collected under anesthesia with ketamine (100 mg/kg) and xylazine (10 mg/kg) via cardiac puncture. Two milliliters of the blood was used to determine hematological parameters (RBC, WBC, Hb, PCV, and the percentages of neutrophils, lymphocytes, and monocytes) with an EDTA anticoagulant (1:9 ratio of anticoagulant to blood). Approximately 5 mL of the blood samples was centrifuged for 15 min at 1000*g*. The resulting serum was aliquoted and stored at −20°C until further processing. Serum protein determination (albumin, globulin, and total protein) was performed using the electrophoresis method. Additionally, biochemical parameters of the liver and kidney (AST, ALT, ALP, creatinine, and urea) were measured using kits and autoanalyzers. Finally, after euthanasia with an overdose of anesthesia, the liver, kidney, and spleen tissues were fixed, sectioned, and stained with hematoxylin and eosin for histopathological studies [21].

### 2.4. Determination of Serum Protein Electrophoresis Pattern

Blood serum analysis was performed using the CAPILLARYS 3 OCTA automated system, and the albumin, globulin, and total protein electrophoresis levels were calculated using the standard curve [23].

### 2.5. Statistical Analysis

Data were analyzed in SPSS Version 26 using one-way ANOVA and Tukey's post hoc test after confirming the normality of the analysis. In cases of nonnormal data, the Kruskal–Wallis and Mann–Whitney tests were used to complete the results. Significance levels of *p* < 0.05, *p* < 0.01, and *p* < 0.001 were considered.

## 3. Results

### 3.1. Investigation of Hematological Parameter Changes

**Figure 1:**
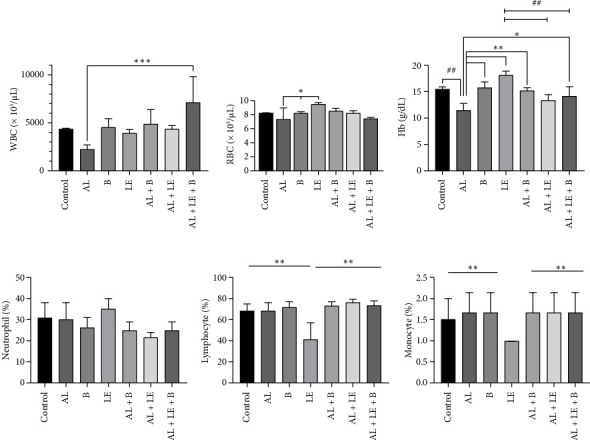
Changes in hematological parameters in different study groups. (a) WBC count (×10^3^/μL); (b) RBC count (×10^6^/μL); (c) Hb (g/dL); (d) neutrophil (%); (e) lymphocyte (%); (f) monocyte (%). Control group without treatment, AL group: lead acetate 1000 ppm in water, B group: broccoli at 300 mg/kg, LE group: levamisole at a dose of 2.5 mg/kg, AL + B group lead acetate in water and broccoli at 300 mg/kg, AL + LE group: lead acetate in water and levamisole at 2.5 mg/kg, and AL + LE + B group: lead acetate in water, broccoli extract, and levamisole at similar doses Significant differences between groups at various levels have been determined with *p* values of ⁣^∗∗∗^ < 0.001, ⁣^∗∗^ < 0.01, and ⁣^∗,#^ < 0.05.

Changes in hematological parameters are depicted in Figure 1. The analysis of blood hematological parameters revealed the highest mean WBC count in the AL + LE + B group (7133.250 ± 269.08) × 10^3^/μL, and the lowest in the AL group (2183.250 ± 536.39) × 10^3^/μL. Additionally, the WBC count in the AE + LE + B group showed a significant increase compared to the AE group (*p* < 0.001).

The highest RBC level in the blood of desert rats was determined in the LE group (9.450 ± 0.33) × 10^6^/μL, and the lowest was in the AL group (7.290 ± 1.59) × 10^6^/μL. This difference between the AL and LE groups was significant (*p* < 0.05).

The lowest Hb level in animal blood was in the AL group (11.50 ± 1.29) g/dL, and the highest was in the LE group (18.20 ± 0.88) g/dL. There was a significant increase in Hb levels in other groups compared to the AL group (*p* < 0.001).

The neutrophil count in the blood of desert rats was the lowest in the AL + LE group (21.5650 ± 2.36)% and the highest in the LE group (35.0000 ± 5.00)%. No significant difference was observed between the different groups (*p* > 0.05).

The lymphocyte and monocyte counts were highest in the AL + LE group, with values of 76.6650 ± 2.69% and 1.66 ± 0.47%, respectively, and lowest in the LE group, with values of 41.0000 ± 8.00 and 1.00 ± 0.00%, respectively. There was a significant difference in the number of lymphocytes between different groups compared to the LE group (*p* < 0.01) (Figure 1).

### 3.2. Changes in the Biochemical Biomarkers of Kidney Function

**Figure 2:**
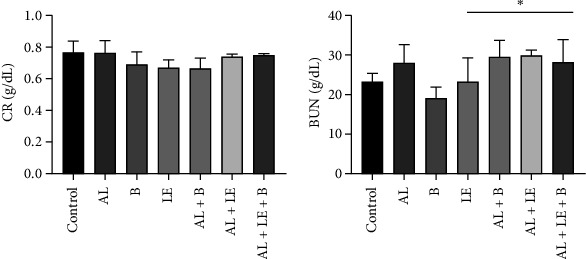
Changes in creatinine and BUN levels in different study groups. (a) Creatinine concentration (g/dL); (b) BUN concentration (g/dL). Control group without treatment, AL group: lead acetate 1000 ppm in water, B group: broccoli at 300 mg/kg, LE group: levamisole at a dose of 2.5 mg/kg, AL + B group lead acetate in water and broccoli at 300 mg/kg, AL + LE group: lead acetate in water and levamisole at 2.5 mg/kg, AL + LE + B group: lead acetate in water, broccoli extract, and levamisole at similar doses. Significant differences between groups at various levels have been determined with *p* values of ⁣^∗^ <  0.05.

The creatinine level in the serum of animals was highest in the AL group (0.768 ± 0.08) g/dL and lowest in the AL + B group (0.67 ± 0.06) g/dL. There was no significant difference between the different groups. Additionally, the BUN level was highest in the AL group and lowest in the B group, and there were significant statistical differences between the B group and control with other groups (*p* < 0.05) (Figure 2).

### 3.3. Changes in the Biochemical Biomarkers of Liver Function

The level of ALP in the liver tissue was highest in the AL group (350.00 ± 0.57) U/L and lowest in the control group (12.90 ± 85.00) U/L. The ALP level significantly decreased in the LE and B groups compared to the AL group (*p* < 0.05). Additionally, in the combination groups AL + LE + B, these values showed a significant decrease compared to the AL group (*p* < 0.001).

**Figure 3:**
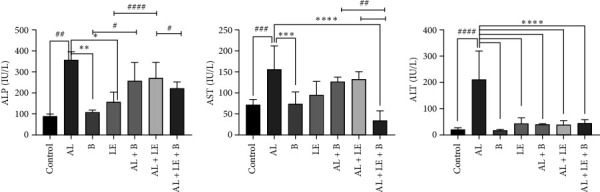
Evaluation of changes in liver enzymes in study groups. (a) ALP (U/L); (b) AST (U/L), (c) ALT (U/L). Control group without treatment, AL group: lead acetate 1000 ppm in water, B group: broccoli at 300 mg/kg, LE group: levamisole at a dose of 2.5 mg/kg, AL + B group lead acetate in water and broccoli at 300 mg/kg, AL + LE group: lead acetate in water and levamisole at 2.5 mg/kg, AL + LE + B group: lead acetate in water, broccoli extract, and levamisole at similar doses. Significant differences between groups at various levels have been determined with *p* values of ⁣^∗∗∗∗^, ^####^< 0.0001, ⁣^∗∗∗^, ^###^0.001, ⁣^∗∗^, ^##^ 0.01, and ⁣^∗^, ^#^0.05.

The determination of liver enzymes is shown in Figure 3. The highest levels of ALT and AST enzymes were observed in the AL group, with values of 156.7000 ± 54.88 U/L and 209.96 U/L ± 108.66 ± U/L, respectively. The lowest levels were found in the AL + LE + B and B groups, with values of 35.4000 ± 21.90 U/L and 17.45 ± 3.92 U/L, respectively. In lead acetate–treated rat liver tissue, the groups treated with methanolic broccoli extract, lead acetate, and levamisole, and methanolic broccoli extract showed a significant decrease compared to the lead acetate–treated group (*p* < 0.05). The AST enzyme in the AL + LE + B group decreased significantly compared to the AL group, reaching levels within the normal range (*p* < 0.0001). The ALT results indicate a significant decrease in enzyme levels in various treatment groups compared to the AL group, with the most significant decrease observed (*p* < 0.0001).

### 3.4. Changes in Serum Protein Biochemistry

**Figure 4:**
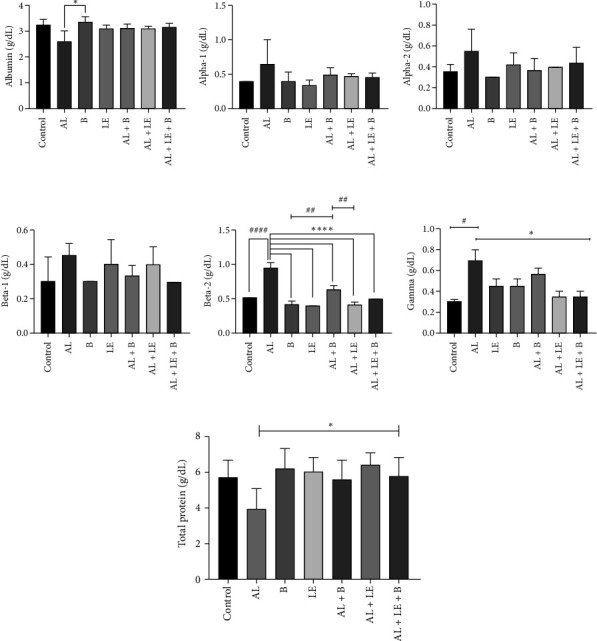
Levels of albumin, different globulins and total protein in the serum of study groups. (a) Albumin concentration (g/dL); (b) Alpha-1(g/dL); (c) Alpha-2 (g/dL); (d) Beta-1 (g/dL); (e) Beta-2 (g/dL); (f) Gamma (g/dL); (g) Total protein (g/dL). Control group without treatment, AL group: lead acetate 1000 ppm in water, B group: broccoli at 300 mg/kg, LE group: levamisole at a dose of 2.5 mg/kg, AL + B group: lead acetate in water and broccoli at 300 mg/kg, AL + LE group: lead acetate in water and levamisole at 2.5 mg/kg, AL + LE + B group: lead acetate in water, broccoli extract, and levamisole at similar doses. Significant differences between groups at various levels have been determined with *p* values of ⁣^∗∗∗∗^, ^####^< 0.0001, ## 0.01, and ⁣^∗^, ^#^0.05.

The level of albumin in the serum protein electrophoresis pattern of rat serum proteins in different groups is presented in Figure 4. The lowest serum albumin concentration was observed in the AL group (2.60 ± 0.424) g/dL, while the highest was in the B group with a mean of 3.35 ± 0.212 g/dL. There was a significant statistical difference between the AL and B groups (*p* < 0.05).

The highest concentration of Alpha-1 globulin in the serum protein electrophoresis pattern was in the AL group (0.35 ± 0.65) g/dL, while the lowest was in the LE group with a concentration of 0.07 ± 0.35 g/dL. Meanwhile, the highest concentration of Alpha-2 globulin was 0.55 ± 0.21 g/dL in the AL group and the lowest was in the B group with a concentration of 0.00 ± 0.30 g/dL. No significant statistical difference was observed between the experimental groups.

Beta-1 showed the highest concentration in the AL group (0.070 ± 0.450) g/dL and the lowest in the B group with a mean of 0.30 ± 0.00 g/dL. There was no significant statistical difference between the groups (*p* < 0.05). The concentration of Beta-2 was highest in the AL group (0.070 ± 0.95) g/dL and lowest in the AL + B group with a value of 0.028 ± 0.41 g/dL. The concentration of Beta-2 in the AL group was significantly different from the other experimental groups (*p* < 0.001).

Additionally, the highest level of gamma globulin was in the AL group at 0.73 ± 0.03 g/dL, and the lowest was in the AL + LE group. There was a significant statistical difference between the AL group and the other experimental groups (*p* < 0.05).

The total protein levels were lowest in the lead-exposed group and highest in those receiving levamisole and broccoli. The initial reduction in total protein levels was reversed in the groups treated with broccoli and levamisole, resulting in higher amounts. However, the differences observed between the groups were not statistically significant (*p* > 0.05).

### 3.5. Albumin-to-Globulin Ratio in Serum of Study Groups

**Figure 5:**
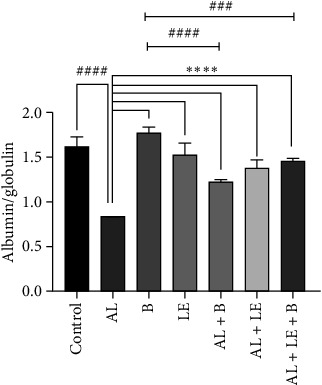
Changes in the albumin-to-globulin ratio in the serum of animals in different experimental groups. Albumin/globulin ratio. Control group without treatment, AL group: lead acetate 1000 ppm in water, B group: broccoli at 300 mg/kg, LE group: levamisole at a dose of 2.5 mg/kg, AL + B group lead acetate in water and broccoli at 300 mg/kg, AL + LE group: lead acetate in water and levamisole at 2.5 mg/kg, AL + LE + B group: lead acetate in water, broccoli extract, and levamisole at similar doses. Significant differences between groups at various levels have been determined with *p* values of ⁣^∗∗∗∗^, ^####^< 0.0001.

The albumin/globulin ratio in the serum protein electrophoresis pattern of animal serum proteins is shown in Figure 5. Accordingly, the highest ratio was in the B group (1.78 ± 0.049), while the lowest was in the AL group (0.85 ± 0.00). This ratio was significantly different from other experimental groups at the level of *p* < 0.0001.

### 3.6. Changes in the Electrophoretic Patterns of Various Serum Proteins Were Observed in Different Groups

**Figure 6:**
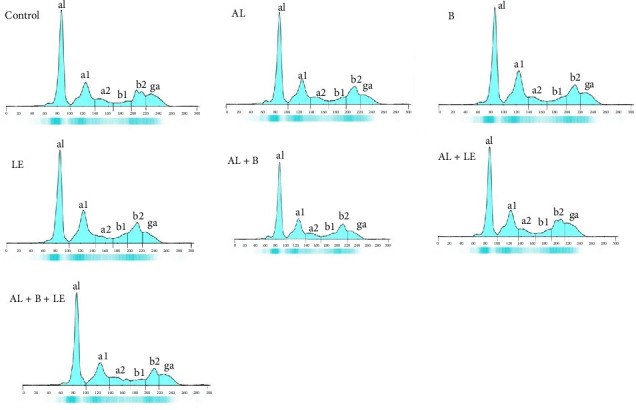
Electrophoresis graph of serum proteins in different experimental groups.

The pattern of changes in serum proteins of the animals under study is displayed in Figure 6. Difference in the concentration of albumin, Alpha-1 and Alpha-2 globulins, Beta-1 and Beta-2 globulins, and gamma globulin in the different treatment groups is shown.

### 3.7. Histopathological Changes in Liver, Spleen, and Kidney Tissues

**Figure 7:**
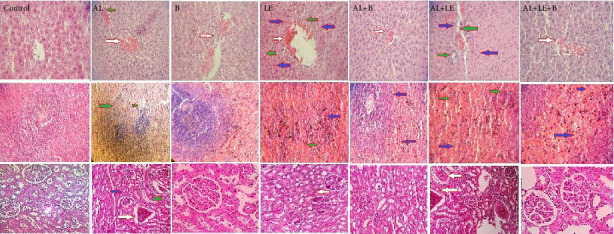
Histopathological slide changes of the studied tissues in different groups (H&E staining, × 100 and 400 magnification). Control group without treatment, AL group: lead acetate 1000 ppm in water, B group: broccoli at 300 mg/kg, LE group: levamisole at a dose of 2.5 mg/kg, AL + B group lead acetate in water and broccoli at 300 mg/kg, AL + LE group: lead acetate in water and levamisole at 2.5 mg/kg, AL + LE + B group: lead acetate in water, broccoli extract, and levamisole at similar doses. The red arrow indicates hyperemia, the blue arrow indicates hemorrhage, and the green arrow indicates inflammatory cells in tissue sections.

In histopathological studies, the highest lesions were observed in the liver and spleen tissues compared to the kidney. Among the evaluated lesions, hemorrhage, accumulation of inflammatory cells, and bleeding showed the highest frequency. Accordingly, the changes were graded, with the highest degree of tissue damage observed in the AL group and the lowest in the AL + B, AL + LE, and AL + LE + B treatment groups. The condition of tissues in the B group was similar to that of the control group, with less grading in liver tissue compared to the control (Table 1, Figure 7).

## 4. Discussion

Exposure to heavy metals like lead, a pervasive pollutant, presents a significant health challenge for human societies [24]. Consequently, utilizing natural and chemical compounds with specialized properties such as antioxidants, anti-inflammatory agents, and immunostimulants to mitigate the detrimental effects of heavy metals on metabolized organs and hematological parameters emerges as a valuable strategy [25]. This study systematically investigates the effects of levamisole and broccoli in experimental lead poisoning.

Hematological analysis revealed that the AL group exhibited the lowest levels of RBCs, leukocytes, and Hb, with a notable increase in their mean levels following treatment with broccoli. Particularly, this increase was more pronounced in the LE group compared to the levamisole and broccoli group in terms of RBC count and Hb levels.

Evaluation of renal biochemical parameters indicated an increase in BUN and creatinine levels in the AL group, with a nonsignificant decrease observed in the treatment groups. However, the administration of broccoli in the B group resulted in a further reduction of creatinine and BUN levels compared to the control group, signifying a more significant improvement in renal function.

Broccoli, rich in flavonoids such as quercetin and kaempferol, phenols, and the potent compound sulforaphane, boasts significant antioxidant capabilities, making it highly effective in combating oxidative stress [26]. Compared to antioxidants like vitamins C and E, broccoli demonstrates more pronounced effects in various interventions [27]. The polyphenols in broccoli exhibit strong antioxidant activity, effectively neutralizing free radicals [28]. Our study showed that broccoli extract significantly improved hematological, biochemical, enzymatic, and tissue-related complications resulting from lead poisoning.

Another study assessed the efficacy of broccoli extract in mitigating arsenic poisoning, with a particular emphasis on its effects on kidney function. The results showed that broccoli extract alleviated lead-induced kidney damage by modulating oxidative stress parameters and enhancing total serum antioxidant capacity [29]. Vega-Galvez et al. further confirmed the anti-inflammatory and antioxidant properties of broccoli, attributing these benefits to its phenolic and flavonoid components [30].

Additionally, Subhi and Al-Okaily highlighted the effectiveness of resveratrol in lowering calcium and phosphorus levels and reducing oxidative damage caused by lead poisoning in rats. Given broccoli's significant calcium content, its potential role in mitigating calcium imbalances associated with lead toxicity should be explored further [31].

Furthermore, another study demonstrated the efficacy of broccoli in arsenic poisoning and its impact on liver and kidney function and damage grades. The herbal combination showed promise in mitigating arsenic poisoning, as evidenced by increased total serum antioxidant capacity and AST and ALT enzyme levels, along with decreased serum creatinine and urea levels. This underscores the extract's positive effect on antioxidant processes and the inhibition of free radicals induced by heavy metals [32].

Levamisole, with its immunomodulatory effects, can be utilized as a stimulating combination in conditions of immune impairments [33]. In a clinical trial measuring workers exposed to lead, their interleukin-4 and gamma-interferon levels were found to increase twofold compared to unexposed individuals [34]. This indicates immune cell suppression in chronic lead exposure, which can potentially increase the risk of cancer.

In another study regarding the performance of N-acetylcysteine in lead poisoning in rats, selenium supplementation with *N*-acetylcysteine was shown to alleviate lead-induced oxidative stress biochemically in blood and tissue, reduce the body burden of lead, and improve molecular changes by compensating for average DNA damage [35].

In recent histopathological findings, the highest levels of lead damage were observed in the liver compared to the kidneys and spleen. Liver-specific enzymes showed a significant increase, and treatment with broccoli and levamisole demonstrated remarkable effectiveness. Other results indicated liver damage manifested by decreased albumin levels and increased globulins, confirming a significant reduction in the albumin-to-globulin ratio in the lead-exposed group compared to other treatment groups in our study [32]. Various factors can contribute to increased globulin levels, including immune system impairments, hemolytic anemia, and cancer. This hemorrhage can be assessed intravascularly or extravascularly (spleen). Changes in hemorrhage and the presence of hemosiderin in the spleen tissue, resulting from Hb breakdown, could be potential causes in our recent study.

The increase in globulins, particularly Beta-2 globulins, in the serum during lead poisoning is due to damage to the kidney glomeruli. Consequently, the kidneys lose their filtering ability, leading to elevated globulin levels in the blood. Additionally, liver damage caused by lead poisoning results in decreased albumin synthesis, lowering its concentration [36].

An increase in gamma globulin levels can indicate immune system weakening, as observed in the lead-exposed group in our recent study. Treatment with levamisole and the combination of broccoli and levamisole effectively reduced this increase. The rise in gamma globulin levels has been shown as a nonspecific marker in the humoral immune system, which can elevate inflammation and infection risks [37].

In light of inflammation induced by lead-related liver damage, supported by pathological findings from our study, the increase in gamma globulin levels is justified [38]. Treatment with levamisole and broccoli strengthens the immune system, controls inflammation, and mitigates the progression of lead poisoning, thereby confirming a decrease in globulin levels, including gamma globulins.

It is noteworthy that liver toxicity occurs through various mechanisms. One of the major mechanisms associated with hepatotoxicity is the formation of free radicals and oxidative stress in the liver [39]. Free radicals and oxidative stress play a significant role in liver toxicity and some other liver diseases. Free radical species can interact with cellular macromolecules such as lipids, proteins, and nucleic acids, ultimately leading to oxidative damage to cell membranes and cellular injury [40]. Various experimental models are used to study the mechanisms of liver toxicity and investigate the potential therapeutic effects of different compounds. The findings of the current study showed that, on the one hand, lead poisoning has led to a significant increase in serum levels of liver enzymes, as expected, and, on the other hand, concurrent treatment under lead poisoning with broccoli extract and levamisole has led to a decrease in liver enzyme levels.

In this regard, quercetin is one of the important flavonoids present in broccoli extract [41]. This substance is also abundantly found in walnut leaves. In a study conducted by Mirkov and colleagues on the hepatoprotective effect of walnut leaf extract, it was shown that the protective effect of the extract is due to the high level of quercetin in it [42]. They demonstrated that quercetin, due to having two adjacent hydroxyl groups, enhances the reductive power and hence the antioxidant power of this substance. Moreover, quercetin protects lymphocytes from damage caused by chemical and toxic substances. This substance increases plasma antioxidant capacity and stabilizes the genome in mice with liver cirrhosis. Additionally, by scavenging free radicals, it prevents genome damage and mutation [43].

Lead poisoning is implicated in oxidative tissue damage due to its ability to decrease the levels of GSH and protein-bound sulfhydryl groups, as well as alter the activity of various antioxidant enzymes indicative of lipid peroxidation [44]. Lead is known to exert toxic effects on membrane structure and function by changing the fatty acid composition, thereby increasing susceptibility to lipid peroxidation. This effect is particularly evident in erythrocyte membranes, as erythrocytes have a high affinity for lead and are more vulnerable to oxidative damage than many other cells [45].

Broccoli, with its strong sulfur-containing antioxidant compounds such as sulforaphane, can be effective in this intervention and in increasing the levels of GSH peroxidase. Meanwhile, another mechanism of lead poisoning can be highlighted through in vitro studies that demonstrated lead treatment of macrophages induces the dysregulation of proinflammatory cytokine production, such as tumor necrosis factor-α (TNF-α), interleukin-1*α* (IL-1α), and IL-6, and promotes the synthesis of Th1 cytokines (interferon [IFN]-*γ* and IL-2) [46]. Considering the properties of levamisole, this drug, with its immunostimulatory effects, can prevent the increase of proinflammatory cytokines.

## 5. Conclusion

The research results indicate that lead poisoning can cause liver, kidney, and spleen damage, with liver tissue damage being more prominent than other tissues. Changes in biochemical parameters, including liver enzymes, and a decrease in albumin levels support this effect. In this regard, treatment with broccoli extract, with its antioxidant, anti-inflammatory, and immune-enhancing effects, could improve lead poisoning conditions. This was evident by a significant reduction in liver enzyme levels and an increase in albumin levels. During treatment with levamisole and broccoli, the performance of these two compounds on the immune system and hematological parameters was more favorable.

Therefore, levamisole medication in inflammatory conditions and immune system impairment, along with broccoli extract as an antioxidant, can be beneficial in preventing heavy metal toxicity, especially lead.

The clinical application of levamisole and broccoli to enhance immune system function, hematological parameters, and hepatic and renal biochemical markers in preventing oxidative damage from heavy metals, especially lead, is the focus of this scientific paper. Evaluating inflammatory parameters and molecular changes in the expression of antioxidant enzyme genes are among the study's limitations recommended for further exploration to complete the research findings.

## Figures and Tables

**Table 1:** Histopathological evaluation of tissue in experimental groups.

	C	AL	B	LE	AL + B	AL + LE	AL + LE + B
Hyperemia	0.00 (0.00–0.40)	2.80 (1.90–3.00) ##, ⁣^∗^	0.00 (0.00–0.02)	0.05 (0.00–0.08)	1.20 (0.5-.02)	1.50 (1.00–2.00)	1.20 (1.00–1.90)
Inflammatory cells	0.00 (0.00–0.00)	3.00 (1.80–3.50) ###, ⁣^∗^	0.00 (0.00–0.00)	0.02 (0.00–0.05)	1.90 (0.50–1.00)	1.50 (0.4–1.60)	1.2 (0.8–1.5)
Hemorrhage	0.00 (0.00–0.00)	1.70 (0.80–1.90) #, ⁣^∗^	0.00 (0.00–0.00)	0.00 (0.00–0.00)	1.00 (0.60–1.50)	1.20 (0.80–1.70)	1.00 (0.6–1.10)

*Note:* Control group without treatment, AL group: lead acetate 1000 ppm in water, B group: broccoli at 300 mg/kg, LE group: levamisole at a dose of 2.5 mg/kg, AL + B group lead acetate in water and broccoli at 300 mg/kg, AL + LE group: lead acetate in water and levamisole at 2.5 mg/kg, AL + LE + B group: lead acetate in water, broccoli extract, and levamisole at similar doses.

Signs indicate significant differences between groups. Significant differences between groups at various levels have been determined with *p* values of ^###^< 0.001, ^##^< 0.01, ⁣^∗^< 0.05.

## Data Availability

The datasets used and/or analyzed during the current study are available from the corresponding author upon reasonable request.
